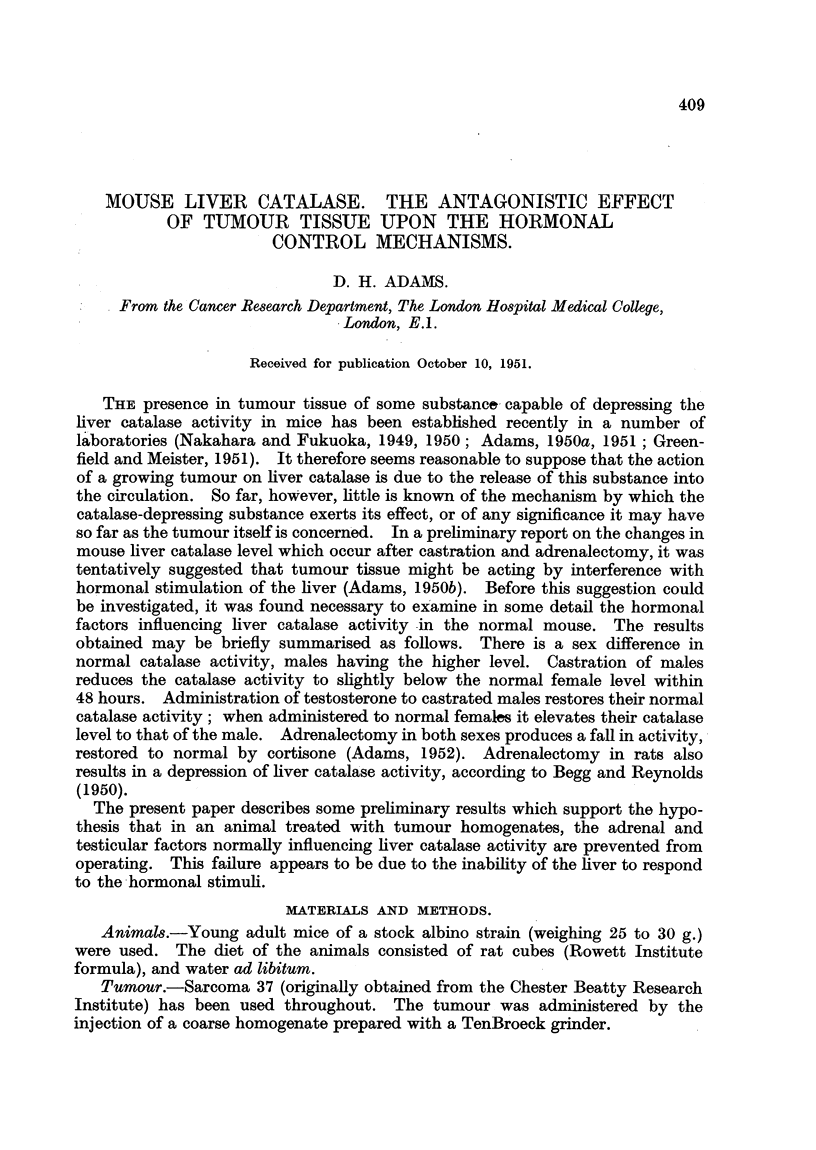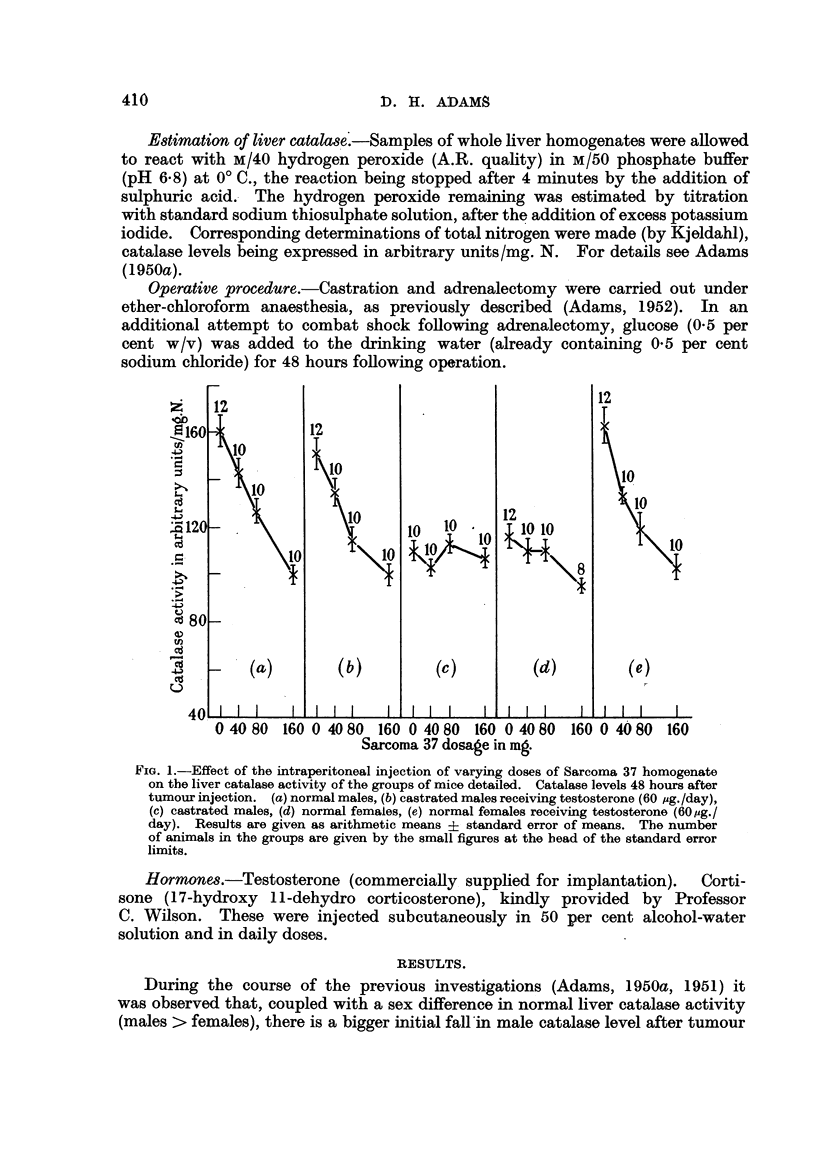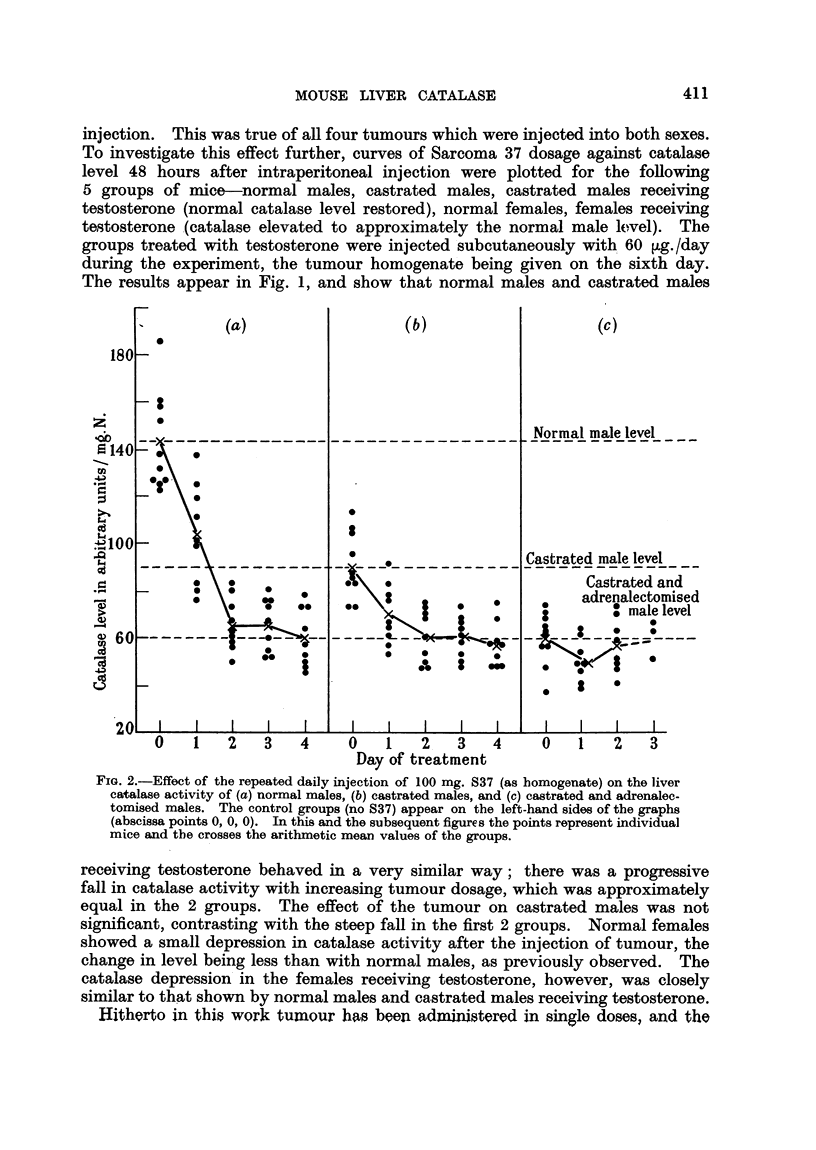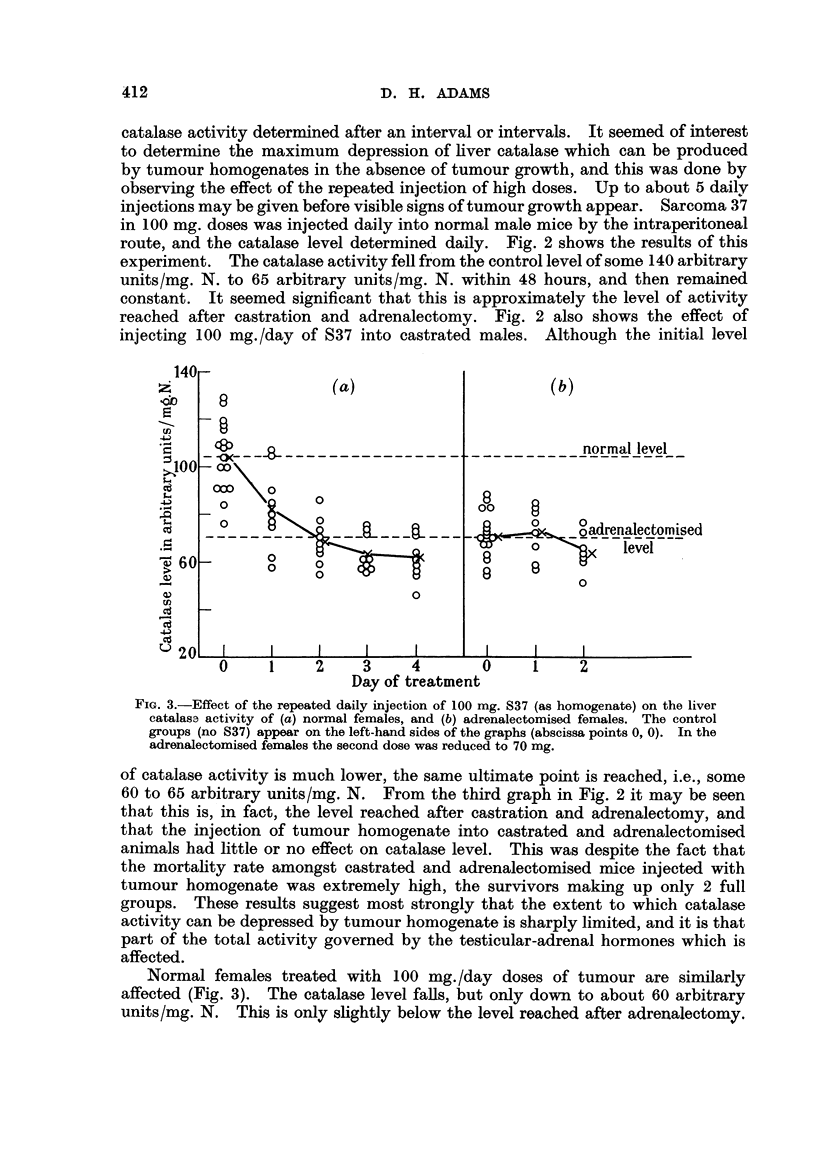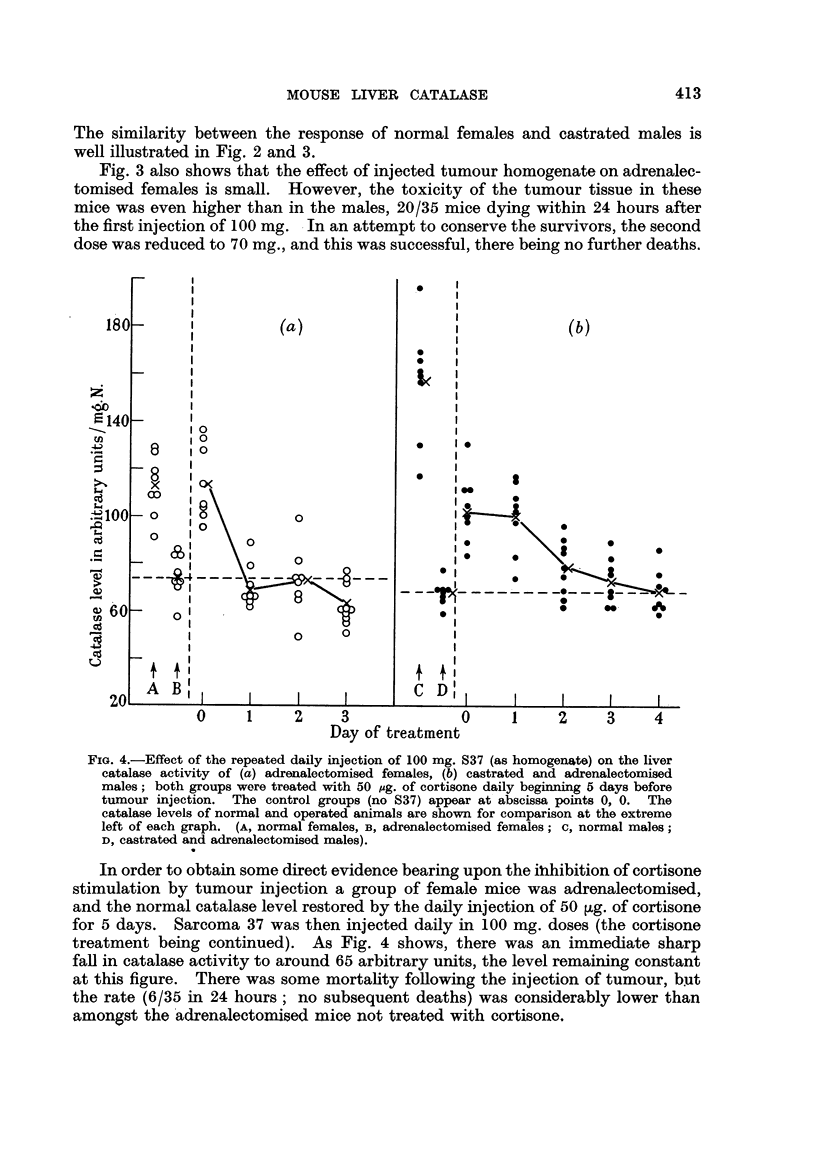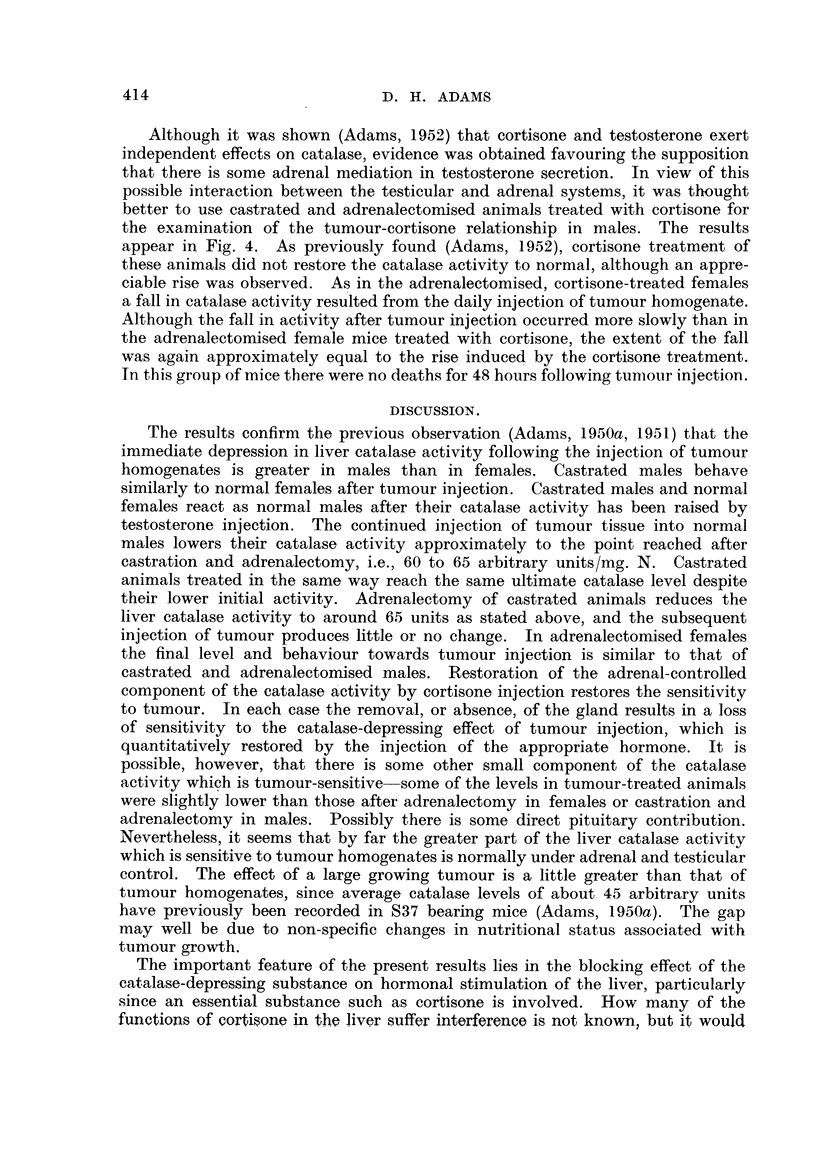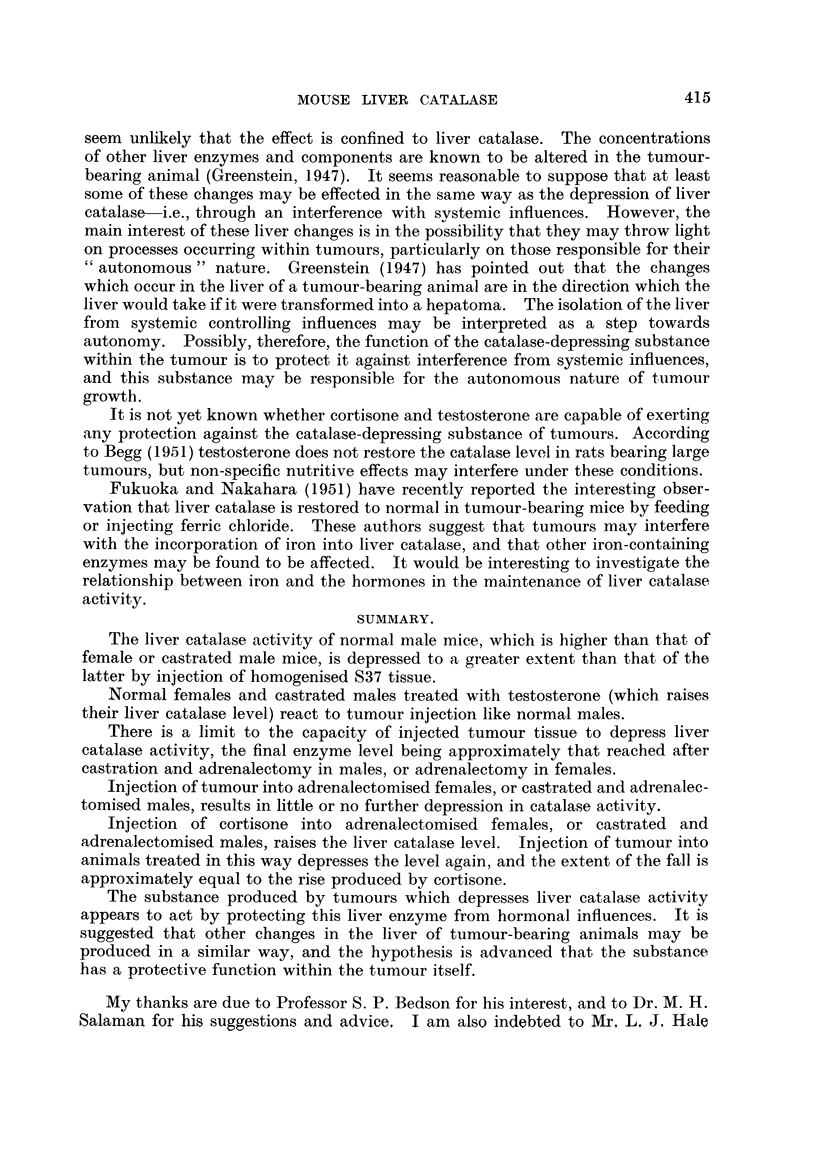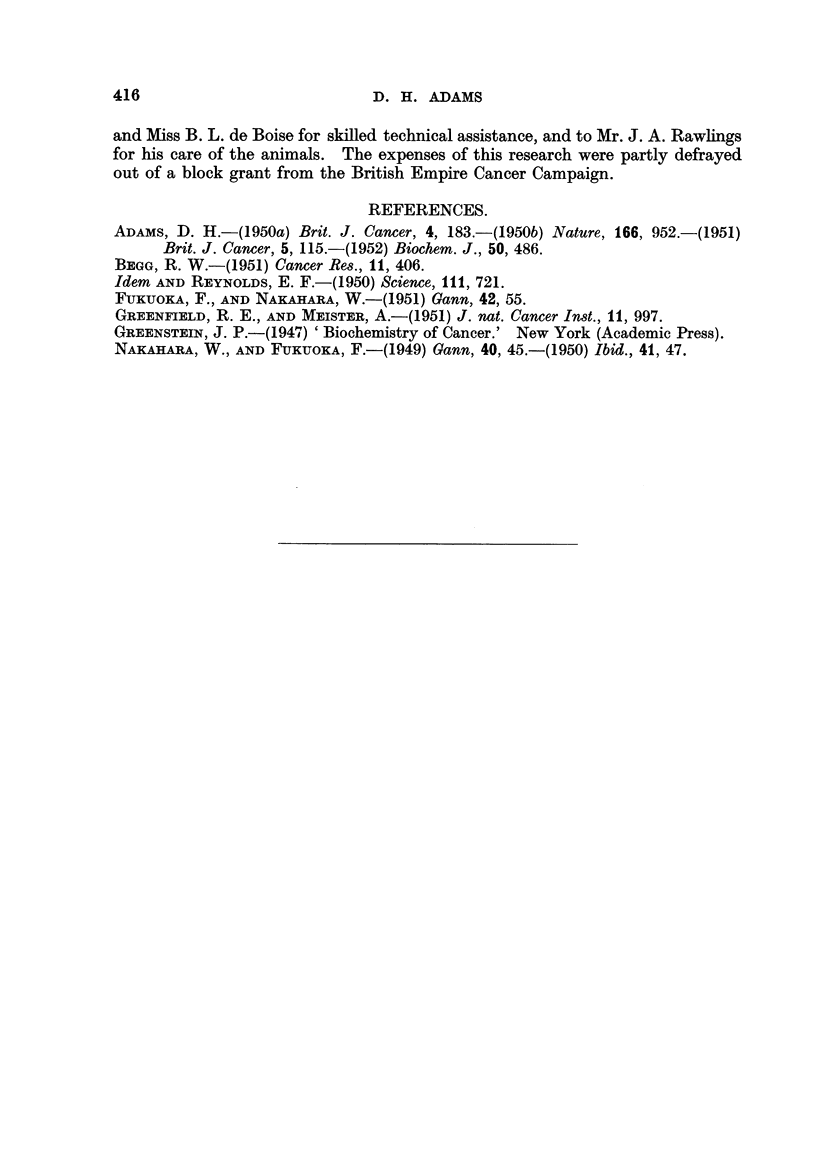# Mouse Liver Catalase. The Antagonistic Effect of Tumour Tissue upon the Hormonal Control Mechanisms

**DOI:** 10.1038/bjc.1951.47

**Published:** 1951-12

**Authors:** D. H. Adams


### MOUSE LIVER CATALASE. THE ANTAGONISTIC EFFECT OF TUMOUR TISSUE UPON THE HORMONAL CONTROL MECHANISMS.

D. H. ADAMS.

*From the Cancer Research Department, The London Hospital Medical College, London, E.1.*

Received for publication October 10, 1951.

THE presence in tumour tissue of some substance capable of depressing the liver catalase activity in mice has been established recently in a number of laboratories (Nakahara and Fukuoka, 1949, 1950; Adams, 1950*a*, 1951; Greenfield and Meister, 1951). It therefore seems reasonable to suppose that the action of a growing tumour on liver catalase is due to the release of this substance into the circulation. So far, however, little is known of the mechanism by which the catalase-depressing substance exerts its effect, or of any significance it may have so far as the tumour itself is concerned. In a preliminary report on the changes in mouse liver catalase level which occur after castration and adrenalectomy, it was tentatively suggested that tumour tissue might be acting by interference with hormonal stimulation of the liver (Adams, 1950*b*). Before this suggestion could be investigated, it was found necessary to examine in some detail the hormonal factors influencing liver catalase activity in the normal mouse. The results obtained may be briefly summarised as follows. There is a sex difference in normal catalase activity, males having the higher level. Castration of males reduces the catalase activity to slightly below the normal female level within 48 hours. Administration of testosterone to castrated males restores their normal catalase activity; when administered to normal females it elevates their catalase level to that of the male. Adrenalectomy in both sexes produces a fall in activity, restored to normal by cortisone (Adams, 1952). Adrenalectomy in rats also results in a depression of liver catalase activity, according to Begg and Reynolds (1950).

The present paper describes some preliminary results which support the hypothesis that in an animal treated with tumour homogenates, the adrenal and testicular factors normally influencing liver catalase activity are prevented from operating. This failure appears to be due to the inability of the liver to respond to the hormonal stimuli.

### MATERIALS AND METHODS.

*Animals.*—Young adult mice of a stock albino strain (weighing 25 to 30 g.) were used. The diet of the animals consisted of rat cubes (Rowett Institute formula), and water *ad libitum*.

*Tumour.*—Sarcoma 37 (originally obtained from the Chester Beatty Research Institute) has been used throughout. The tumour was administered by the injection of a coarse homogenate prepared with a TenBroeck grinder.

*Estimation of liver catalase.*—Samples of whole liver homogenates were allowed to react with M/40 hydrogen peroxide (A.R. quality) in M/50 phosphate buffer (pH 6·8) at 0° C., the reaction being stopped after 4 minutes by the addition of sulphuric acid. The hydrogen peroxide remaining was estimated by titration with standard sodium thiosulphate solution, after the addition of excess potassium iodide. Corresponding determinations of total nitrogen were made (by Kjeldahl), catalase levels being expressed in arbitrary units/mg. N. For details see Adams (1950*a*).

*Operative procedure.*—Castration and adrenalectomy were carried out under ether-chloroform anaesthesia, as previously described (Adams, 1952). In an additional attempt to combat shock following adrenalectomy, glucose (0·5 per cent w/v) was added to the drinking water (already containing 0·5 per cent sodium chloride) for 48 hours following operation.

FIG. 1.—Effect of the intraperitoneal injection of varying doses of Sarcoma 37 homogenate on the liver catalase activity of the groups of mice detailed. Catalase levels 48 hours after tumour injection. (*a*) normal males, (*b*) castrated males receiving testosterone (60 $\mu$g./day), (*c*) castrated males, (*d*) normal females, (*e*) normal females receiving testosterone (60 $\mu$g./day). Results are given as arithmetic means $\pm$ standard error of means. The number of animals in the groups are given by the small figures at the head of the standard error limits.

*Hormones.*—Testosterone (commercially supplied for implantation). Cortisone (17-hydroxy 11-dehydro corticosterone), kindly provided by Professor C. Wilson. These were injected subcutaneously in 50 per cent alcohol-water solution and in daily doses.

### RESULTS.

During the course of the previous investigations (Adams, 1950*a*, 1951) it was observed that, coupled with a sex difference in normal liver catalase activity (males > females), there is a bigger initial fall in male catalase level after tumour injection. This was true of all four tumours which were injected into both sexes. To investigate this effect further, curves of Sarcoma 37 dosage against catalase level 48 hours after intraperitoneal injection were plotted for the following 5 groups of mice—normal males, castrated males, castrated males receiving testosterone (normal catalase level restored), normal females, females receiving testosterone (catalase elevated to approximately the normal male level). The groups treated with testosterone were injected subcutaneously with 60 μg./day during the experiment, the tumour homogenate being given on the sixth day. The results appear in Fig. 1, and show that normal males and castrated males

FIG. 2.—Effect of the repeated daily injection of 100 mg. S37 (as homogenate) on the liver catalase activity of (*a*) normal males, (*b*) castrated males, and (c) castrated and adrenalectomised males. The control groups (no S37) appear on the left-hand sides of the graphs (abscissa points 0, 0, 0). In this and the subsequent figures the points represent individual mice and the crosses the arithmetic mean values of the groups.

receiving testosterone behaved in a very similar way ; there was a progressive fall in catalase activity with increasing tumour dosage, which was approximately equal in the 2 groups. The effect of the tumour on castrated males was not significant, contrasting with the steep fall in the first 2 groups. Normal females showed a small depression in catalase activity after the injection of tumour, the change in level being less than with normal males, as previously observed. The catalase depression in the females receiving testosterone, however, was closely similar to that shown by normal males and castrated males receiving testosterone.

Hitherto in this work tumour has been administered in single doses, and the catalase activity determined after an interval or intervals. It seemed of interest to determine the maximum depression of liver catalase which can be produced by tumour homogenates in the absence of tumour growth, and this was done by observing the effect of the repeated injection of high doses. Up to about 5 daily injections may be given before visible signs of tumour growth appear. Sarcoma 37 in 100 mg. doses was injected daily into normal male mice by the intraperitoneal route, and the catalase level determined daily. Fig. 2 shows the results of this experiment. The catalase activity fell from the control level of some 140 arbitrary units/mg. N. to 65 arbitrary units/mg. N. within 48 hours, and then remained constant. It seemed significant that this is approximately the level of activity reached after castration and adrenalectomy. Fig. 2 also shows the effect of injecting 100 mg./day of S37 into castrated males. Although the initial level

FIG. 3.—Effect of the repeated daily injection of 100 mg. S37 (as homogenate) on the liver catalase activity of (*a*) normal females, and (*b*) adrenalectomised females. The control groups (no S37) appear on the left-hand sides of the graphs (abscissa points 0, 0). In the adrenalectomised females the second dose was reduced to 70 mg.

of catalase activity is much lower, the same ultimate point is reached, i.e., some 60 to 65 arbitrary units/mg. N. From the third graph in Fig. 2 it may be seen that this is, in fact, the level reached after castration and adrenalectomy, and that the injection of tumour homogenate into castrated and adrenalectomised animals had little or no effect on catalase level. This was despite the fact that the mortality rate amongst castrated and adrenalectomised mice injected with tumour homogenate was extremely high, the survivors making up only 2 full groups. These results suggest most strongly that the extent to which catalase activity can be depressed by tumour homogenate is sharply limited, and it is that part of the total activity governed by the testicular-adrenal hormones which is affected.

Normal females treated with 100 mg./day doses of tumour are similarly affected (Fig. 3). The catalase level falls, but only down to about 60 arbitrary units/mg. N. This is only slightly below the level reached after adrenalectomy.

The similarity between the response of normal females and castrated males is well illustrated in Fig. 2 and 3.

Fig. 3 also shows that the effect of injected tumour homogenate on adrenalectomised females is small. However, the toxicity of the tumour tissue in these mice was even higher than in the males, 20/35 mice dying within 24 hours after the first injection of 100 mg. In an attempt to conserve the survivors, the second dose was reduced to 70 mg., and this was successful, there being no further deaths.

FIG. 4.—Effect of the repeated daily injection of 100 mg. S37 (as homogenate) on the liver catalase activity of (*a*) adrenalectomised females, (*b*) castrated and adrenalectomised males; both groups were treated with 50 $\mu$g. of cortisone daily beginning 5 days before tumour injection. The control groups (no S37) appear at abscissa points 0, 0. The catalase levels of normal and operated animals are shown for comparison at the extreme left of each graph. (A, normal females, B, adrenalectomised females; C, normal males; D, castrated and adrenalectomised males).

In order to obtain some direct evidence bearing upon the inhibition of cortisone stimulation by tumour injection a group of female mice was adrenalectomised, and the normal catalase level restored by the daily injection of 50 $\mu$g. of cortisone for 5 days. Sarcoma 37 was then injected daily in 100 mg. doses (the cortisone treatment being continued). As Fig. 4 shows, there was an immediate sharp fall in catalase activity to around 65 arbitrary units, the level remaining constant at this figure. There was some mortality following the injection of tumour, but the rate (6/35 in 24 hours; no subsequent deaths) was considerably lower than amongst the adrenalectomised mice not treated with cortisone.

Although it was shown (Adams, 1952) that cortisone and testosterone exert independent effects on catalase, evidence was obtained favouring the supposition that there is some adrenal mediation in testosterone secretion. In view of this possible interaction between the testicular and adrenal systems, it was thought better to use castrated and adrenalectomised animals treated with cortisone for the examination of the tumour-cortisone relationship in males. The results appear in Fig. 4. As previously found (Adams, 1952), cortisone treatment of these animals did not restore the catalase activity to normal, although an appreciable rise was observed. As in the adrenalectomised, cortisone-treated females a fall in catalase activity resulted from the daily injection of tumour homogenate. Although the fall in activity after tumour injection occurred more slowly than in the adrenalectomised female mice treated with cortisone, the extent of the fall was again approximately equal to the rise induced by the cortisone treatment. In this group of mice there were no deaths for 48 hours following tumour injection.

### DISCUSSION.

The results confirm the previous observation (Adams, 1950*a*, 1951) that the immediate depression in liver catalase activity following the injection of tumour homogenates is greater in males than in females. Castrated males behave similarly to normal females after tumour injection. Castrated males and normal females react as normal males after their catalase activity has been raised by testosterone injection. The continued injection of tumour tissue into normal males lowers their catalase activity approximately to the point reached after castration and adrenalectomy, i.e., 60 to 65 arbitrary units/mg. N. Castrated animals treated in the same way reach the same ultimate catalase level despite their lower initial activity. Adrenalectomy of castrated animals reduces the liver catalase activity to around 65 units as stated above, and the subsequent injection of tumour produces little or no change. In adrenalectomised females the final level and behaviour towards tumour injection is similar to that of castrated and adrenalectomised males. Restoration of the adrenal-controlled component of the catalase activity by cortisone injection restores the sensitivity to tumour. In each case the removal, or absence, of the gland results in a loss of sensitivity to the catalase-depressing effect of tumour injection, which is quantitatively restored by the injection of the appropriate hormone. It is possible, however, that there is some other small component of the catalase activity which is tumour-sensitive—some of the levels in tumour-treated animals were slightly lower than those after adrenalectomy in females or castration and adrenalectomy in males. Possibly there is some direct pituitary contribution. Nevertheless, it seems that by far the greater part of the liver catalase activity which is sensitive to tumour homogenates is normally under adrenal and testicular control. The effect of a large growing tumour is a little greater than that of tumour homogenates, since average catalase levels of about 45 arbitrary units have previously been recorded in S37 bearing mice (Adams, 1950*a*). The gap may well be due to non-specific changes in nutritional status associated with tumour growth.

The important feature of the present results lies in the blocking effect of the catalase-depressing substance on hormonal stimulation of the liver, particularly since an essential substance such as cortisone is involved. How many of the functions of cortisone in the liver suffer interference is not known, but it would seem unlikely that the effect is confined to liver catalase. The concentrations of other liver enzymes and components are known to be altered in the tumour-bearing animal (Greenstein, 1947). It seems reasonable to suppose that at least some of these changes may be effected in the same way as the depression of liver catalase—i.e., through an interference with systemic influences. However, the main interest of these liver changes is in the possibility that they may throw light on processes occurring within tumours, particularly on those responsible for their "autonomous" nature. Greenstein (1947) has pointed out that the changes which occur in the liver of a tumour-bearing animal are in the direction which the liver would take if it were transformed into a hepatoma. The isolation of the liver from systemic controlling influences may be interpreted as a step towards autonomy. Possibly, therefore, the function of the catalase-depressing substance within the tumour is to protect it against interference from systemic influences, and this substance may be responsible for the autonomous nature of tumour growth.

It is not yet known whether cortisone and testosterone are capable of exerting any protection against the catalase-depressing substance of tumours. According to Begg (1951) testosterone does not restore the catalase level in rats bearing large tumours, but non-specific nutritive effects may interfere under these conditions.

Fukuoka and Nakahara (1951) have recently reported the interesting observation that liver catalase is restored to normal in tumour-bearing mice by feeding or injecting ferric chloride. These authors suggest that tumours may interfere with the incorporation of iron into liver catalase, and that other iron-containing enzymes may be found to be affected. It would be interesting to investigate the relationship between iron and the hormones in the maintenance of liver catalase activity.

### SUMMARY.

The liver catalase activity of normal male mice, which is higher than that of female or castrated male mice, is depressed to a greater extent than that of the latter by injection of homogenised S37 tissue.

Normal females and castrated males treated with testosterone (which raises their liver catalase level) react to tumour injection like normal males.

There is a limit to the capacity of injected tumour tissue to depress liver catalase activity, the final enzyme level being approximately that reached after castration and adrenalectomy in males, or adrenalectomy in females.

Injection of tumour into adrenalectomised females, or castrated and adrenalectomised males, results in little or no further depression in catalase activity.

Injection of cortisone into adrenalectomised females, or castrated and adrenalectomised males, raises the liver catalase level. Injection of tumour into animals treated in this way depresses the level again, and the extent of the fall is approximately equal to the rise produced by cortisone.

The substance produced by tumours which depresses liver catalase activity appears to act by protecting this liver enzyme from hormonal influences. It is suggested that other changes in the liver of tumour-bearing animals may be produced in a similar way, and the hypothesis is advanced that the substance has a protective function within the tumour itself.

My thanks are due to Professor S. P. Bedson for his interest, and to Dr. M. H. Salaman for his suggestions and advice. I am also indebted to Mr. L. J. Hale and Miss B. L. de Boise for skilled technical assistance, and to Mr. J. A. Rawlings for his care of the animals. The expenses of this research were partly defrayed out of a block grant from the British Empire Cancer Campaign.

### REFERENCES.

ADAMS, D. H.—(1950*a*) *Brit. J. Cancer*, **4**, 183.—(1950*b*) *Nature*, **166**, 952.—(1951) *Brit. J. Cancer*, **5**, 115.—(1952) *Biochem. J.*, **50**, 486.
BEGG, R. W.—(1951) *Cancer Res.*, **11**, 406.
*Idem* AND REYNOLDS, E. F.—(1950) *Science*, **111**, 721.
FUKUOKA, F., AND NAKAHARA, W.—(1951) *Gann*, **42**, 55.
GREENFIELD, R. E., AND MEISTER, A.—(1951) *J. nat. Cancer Inst.*, **11**, 997.
GREENSTEIN, J. P.—(1947) ' Biochemistry of Cancer.' New York (Academic Press).
NAKAHARA, W., AND FUKUOKA, F.—(1949) *Gann*, **40**, 45.—(1950) *Ibid.*, **41**, 47.